# Frontotemporal Dementia-Parkinsonism Due to *MAPT* Gene Variant Presenting with Rest and Action Tremor

**DOI:** 10.5334/tohm.804

**Published:** 2023-09-21

**Authors:** Shakya Bhattacharjee, Christopher Kobylecki

**Affiliations:** 1 Department of Neurology, Manchester Centre for Clinical Neurosciences, Northern Care Alliance NHS Foundation Trust, Stott Lane, Salford, M6 8HD, UK; 2 Department of Neurology, Manchester Centre for Clinical Neurosciences, Northern Care Alliance NHS Foundation Trust, Manchester Academic Health Sciences Centre, University of Manchester, Manchester, UK

**Keywords:** rest tremor, MAPT, mutation, dementia, FTD

## Abstract

A 50-year-old male presented with a four-year history of gradually progressive rest tremor in the distal right lower limb and then spreading to the left lower limb in last 10-12 months. He developed right arm rest and action tremor two years later. Magnetic resonance imaging scans showed progressive frontotemporal and asymmetrical mesial temporal atrophy. Genetic testing revealed a heterozygous c.915+16C>T pathogenic variant in intron 9 of the *MAPT* gene. Presentation with rest tremor should not exclude frontotemporal dementia-parkinsonism due to a *MAPT* variant as a differential diagnosis though rest tremor is a rare presentation.

A 50-year-old English Caucasian male presented with a four-year history of gradually progressive rest tremor in the distal right lower limb and then spreading to the left lower limb in last 10–12 months. He noticed no slowness or stiffness then. He developed right arm rest and action tremor two years after the lower limb rest tremor and struggled to hold cups even with both hands. He had a 3-year history of progressive imbalance and unprovoked falls without a history of gait freezing. His dexterity was also affected with difficulty dressing, shaving, and writing.

Two years following the onset of the lower limb tremor, he developed short term memory difficulties, progressive forgetfulness, and tendency to lose track in conversation. He required prompting with activities of daily living. He reported word finding difficulty. He became increasingly apathetic, lacked empathy and emotional warmth but had no frank disinhibition. He performed stereotypical movements like opening and closing his hand and picking at his skin that led to bleeding at times. There was no history of visual problems, dry eyes, or photophobia.

He underwent resection of a left frontal meningioma 28 years ago with subsequent focal seizures with secondary generalisation well controlled with levetiracetam and lacosamide. He tried propranolol up to 80 mg twice daily for nearly two years with little response to tremor. Primidone was not considered in view of his worsening cognition. Levetiracetam probably did not contribute to his mood changes as he had been on levetiracetam for more than 8–9 years before his mood problem developed. Moreover, his levetiracetam was reduced from 1500 mg twice daily to 1000 mg twice daily but that made no difference to his mood or behaviour. As his seizures remained well controlled with levetiracetam 1000 mg twice daily and lacosamide 250 mg twice daily we did not reduce levetiracetam any further. Electroencephalography performed six months prior revealed no epileptiform activity.

His mother had developed self-neglect and disinhibited behaviour without parkinsonian signs at the age of 52 years. She was diagnosed with frontal lobe dementia but declined genetic testing and died when 60 years old. His maternal uncle and grandmother also had young onset dementia with marked behavioural disturbance and were institutionalised because of this.

Neurological examination revealed reduced facial expression and blink rate with slow but normal vertical saccadic amplitude. His speech was slow, hypophonic and lacked verbal fluency. He had mild axial and four limb rigidity. There was rest tremor of proximal and distal parts of both lower limbs (frequency 4–6 Hz/sec) and predominantly distal right upper limb, which intermittently re-emerged on posture holding. He showed action tremor when pouring water between two cups. (Video 1) There was mild right-sided bradykinesia of finger tapping and pronation-supination movement. He took multiple steps backwards on the pull test. Montreal Cognitive Assessment Score was 21/30 with impairment in delayed recall, verbal fluency, and abstraction.

**Video 1:** **Video shows the clinical features of the patient with MAPT mutation.** Showing **(A)** slowing or horizontal and vertical saccades. **(B)** asymmetric rest tremor of upper and lower limbs with re-emergence and position specificity, and mild right-sided bradykinesia of upper and lower limbs. **(C)** Worsening of tremor when pouring water between two cups. (written consent was obtained from the patients for the video to be submitted/published if accepted in this journal).

Magnetic resonance imaging (MRI) scans showed progressive frontotemporal and mesial temporal atrophy (left > right) as well as stable post-operative surgical changes. (Figure 1A to 1F). There was no significant midbrain atrophy (Figure 2). Genetic testing revealed a heterozygous c.915 + 16C > T pathogenic variant in intron 9 of the microtubule-associated protein tau (*MAPT*) gene. He was diagnosed as autosomal dominant frontotemporal dementia (FTD) with parkinsonism secondary to *MAPT* variant. He showed no response to levodopa up to 450 mg daily and developed progressively impaired mobility and worsening cognitive disturbance over time. He felt levodopa worsened his lower limb heaviness, so dose was not increased any further.

**Figure 1:**
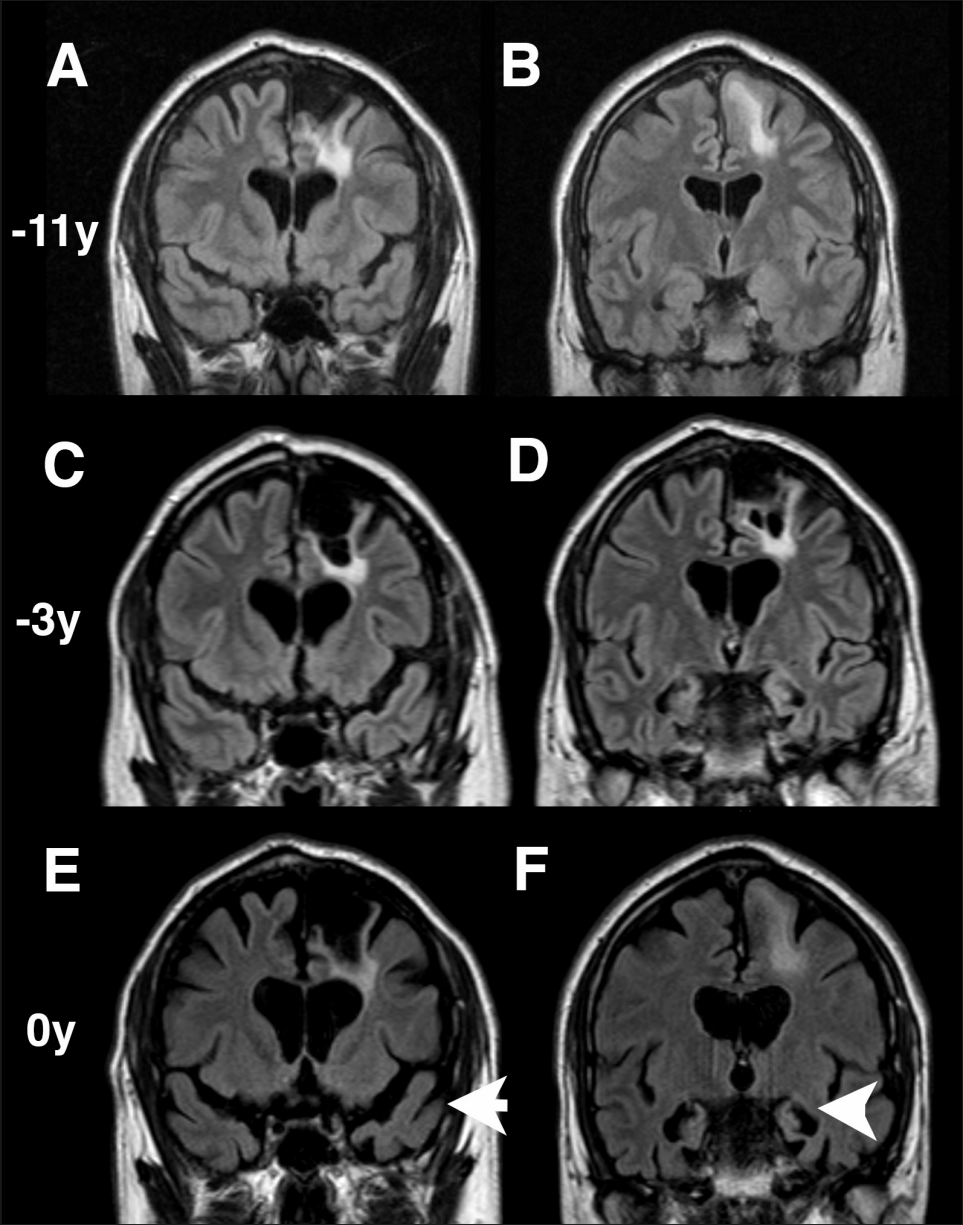
Serial coronal magnetic resonance fluid attenuated inversion recovery images taken at different time points relative to the clinical presentation. **A, C, E** show anterior frontotemporal regions and **B, D, F** show mesial temporal structures. Progressive atrophy of anterior temporal (arrow) and mesial temporal lobe structures (arrowhead) is demonstrated. Left frontal lobe changes secondary to meningioma surgery are also seen.

**Figure 2:**
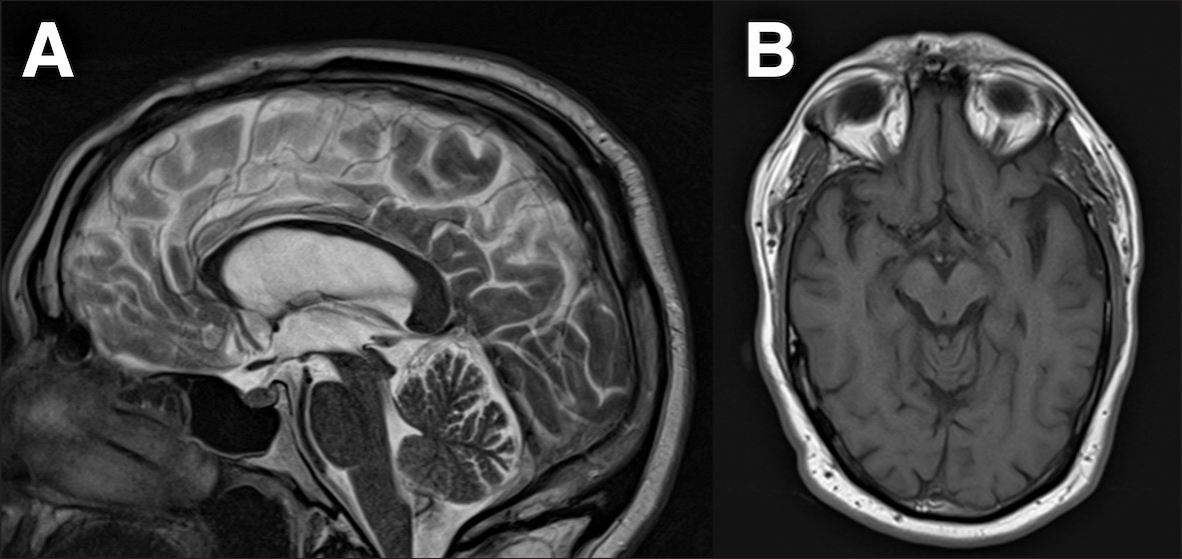
**A:** Sagittal T2-weighted magnetic resonance imaging taken at the time of clinical presentation shows no evidence of hummingbird sign. **B:** Axial T1-weighted MR at the same time point does not show midbrain atrophy, anteroposterior midbrain diameter = 15 mm.

*MAPT, C9orf72* and progranulin (*GRN*) variants are common genetic causes of familial FTD and may also cause parkinsonism [1]. Whereas parkinsonism is a common clinical feature of *MAPT-*associated FTD, marked rest tremor as the presenting feature, as in our patient, is atypical [1]. *MAPT-*associated parkinsonism typically presents as a bilateral akinetic-rigid syndrome, whereas prominent and early rest tremor appears to be a rare feature though lower limb rest tremor was reported in FTD due to *C9ORF72* variants [1,2]. Our patient also had a limited response to levodopa, in particular showing no beneficial effect on tremor. Imaging changes seen in our patient were typical of *MAPT* gene variants and can be seen to be present at and probably before the onset of motor symptoms.

Since rest and action tremor in upper limbs developed simultaneously, we did not feel essential tremor was likely as early rest tremor is unusual in essential tremor though concomitant essential tremor was possible. Rest tremor is rarely described as an early presenting feature in *MAPT-*associated parkinsonism. Hirschbichler, et al reported one patient with a family history of dementia who developed early unilateral rest tremor and bradykinesia and was found to have a c.1216C > T *MAPT* variant [3]. Reed et al reported bilateral upper limb rest and action tremor at an advanced stage in one patient of 15 subsequently confirmed to have c.1216C > T (R406W) *MAPT* variant [4,5]. Tacik and colleagues reported tremor in three of six patients with globular glial tauopathy due to c.951G > C, p.K317N *MAPT* variant, but it occurred later in the disease course whereas all patients had cognitive-behavioural presentations [6]. The same group reported parkinsonism and hand tremor co-occurring with behavioural presentation in one of eight patients with *MAPT* p.P301L (c.902C > T) variant [7]. The variant we report here is well-described in FTD-parkinsonism cases from multiple regions including Wales and North-west England, and has been proven pathogenic [5,8,9].

Our patient had a constellation of symptoms including falls and impaired vertical saccades suggestive of an overlap with Richardson syndrome, a frequent feature of *MAPT* associated disease [1]. The combination with rest tremor and frontal cognitive-behavioural disturbance reinforce the concept that clinical features of genetic FTLD disorders are on a continuum rather than categorical [10]. Accepting the spectral nature of the FTD makes it easier to search for the presence of any parkinsonian signs like rest tremor, understand the pathophysiology; and to guide clinical management and design future clinical trials for FTD. Presentation with rest tremor should not exclude FTD-parkinsonism due to *MAPT* variant as a differential diagnosis.

## Additional File

The additional file for this article can be found as follows:

10.5334/tohm.804.s1 Supplementary figure. Axial and sagittal MRI views of midbrain and cerebellum showing no major changes when compared to 11 years and 3 years from the most recent scan.
