# Supplementary figures and images for: Frontotemporal Dementia-Parkinsonism Due to MAPT Gene Variant Presenting with Rest and Action Tremor
Source: Tremor Other Hyperkinet Mov (N Y). 2023 Sep 21;13:35. doi: 10.5334/tohm.804 (PMC10516137; doi:10.5334/tohm.804)

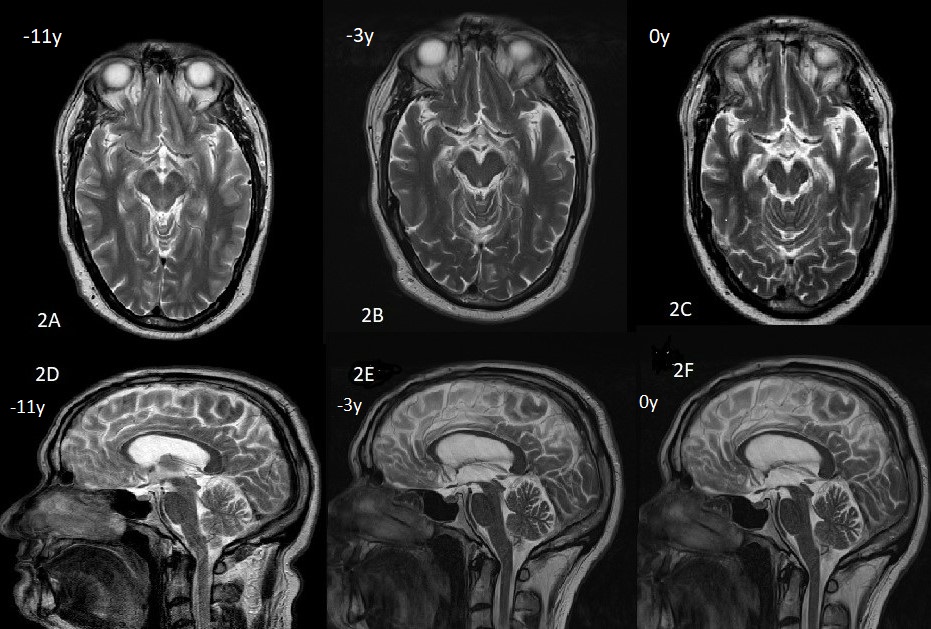

Supplement: Supplementary figure. — Axial and sagittal MRI views of midbrain and cerebellum showing no major changes when compared to 11 years and 3 years from the most recent scan. [file tohm-13-1-804-s1.jpg]
